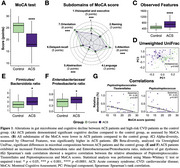# Acute coronary syndrome patients exhibit cognitive impairments and gut dysbiosis compared to high cardiovascular risk patients

**DOI:** 10.1002/alz70857_100074

**Published:** 2025-12-25

**Authors:** Narueporn Pagowong, Kanokphong Suparan, Chanon Kunasol, Chotrawee Piriyakunthorn, Adivitch Sripusanapan, Nutnicha Suntornlekha, Krit Leemasawat, Pannipa Suwannasom, Nipon Chattipakorn, Siriporn C Chattipakorn

**Affiliations:** ^1^ Center of Excellence in Cardiac Electrophysiology Research, Chiang Mai University, Chiang Mai, Thailand; ^2^ Neurophysiology Unit, Cardiac Electrophysiology Research and Training Center, Faculty of Medicine, Chiang Mai University, Chiang Mai, Thailand; ^3^ Department of Physiology, Faculty of Medicine, Chiang Mai University, Chiang Mai, Thailand; ^4^ Immunology Unit, Department of Microbiology, Faculty of Medicine, Chiang Mai University, Chiang Mai, Thailand; ^5^ Division of Cardiovascular Diseases, Department of Medicine, Faculty of Medicine, Chiang Mai University, Chiang Mai, Thailand; ^6^ Department of Oral Biology and Diagnostic Sciences, Faculty of Dentistry, Chiang Mai University, Chiang Mai, Thailand

## Abstract

**Background:**

Previous studies have shown that gut dysbiosis correlates with cognitive impairments in both animal models and clinical settings. Additionally, alterations in gut microbiota have been linked to acute coronary syndrome (ACS). However, the relationship between gut microbiota changes and cognitive outcomes in recent ACS patients remains poorly understood. The present study aims to investigate these changes in gut microbiota and cognitive function in recent ACS patients, comparing them to individuals with high cardiovascular (CV) risks.

**Method:**

The present study enrolled 50 hemodynamically stable ACS patients who experienced myocardial infarction within the past 24 hours, along with 42 patients with high CV risks who served as a control group. This study received approval from the Ethics Committee of the Faculty of Medicine, Chiang Mai University. The Montreal Cognitive Assessment (MoCA) was used to evaluate neurocognitive function. Fecal samples were collected for gut microbiome analysis via 16S rRNA next‐generation sequencing.

**Results:**

Baseline characteristics between stable ACS patients and the control group were significant differences in genders, smoking status, alcohol consumption and some underlying conditions including hypertension and dyslipidemia. Recent ACS patients exhibited a significant decline in MoCA scores (Figure 1A), with lower scores across all subdomains than the control group (Figure 1B). Additionally, recent ACS patients showed gut dysbiosis, evidenced by an increased diversity in the gut microbiota (Figure 1C) and significant differences in microbial composition (Figure 1D) relative to the control group. Notably, recent ACS patients showed mark increases in the Firmicutes/Bacteroidota and *Enterobacteriaceae*/Proteobacteria ratios, suggesting further gut dysbiosis (Figures 1E and 1F). A negative correlation was observed between certain differential taxa—such as *Peptostreptococcales‐Tissierellales* and *Peptostreptococcus*—and MoCA scores in recent ACS patients (Figure 1G).

**Conclusion:**

Our findings suggest an association between alterations in gut microbiota and cognitive impairments in recent ACS patients. Specifically, the presence of *Peptostreptococcales‐Tissierellales* and *Peptostreptococcus* may serve as potential biomarkers for cognitive impairmentsin this population.